# Managing Medication Cost Burden: A Qualitative Study Exploring Experiences of People with Disabilities in Canada

**DOI:** 10.3390/ijerph16173066

**Published:** 2019-08-23

**Authors:** Shikha Gupta, Mary Ann McColl, Sara J.T. Guilcher, Karen Smith

**Affiliations:** 1 School of Rehabilitation Therapy, Queen’s University, Kingston, ON K7L 3N6, Canada; 2 Leslie Dan Faculty of Pharmacy, University of Toronto, Toronto, ON M5S 3M2, Canada; 3 Department of Physical Medicine and Rehabilitation, School of Medicine, Queen’s University, Kingston, ON K7L 3N6, Canada

**Keywords:** medication cost, spinal cord injury, treatment burden, disability, Canada

## Abstract

Despite the abundant literature on the burden of rising costs of prescription medications, there is limited research to explore how these costs affect people and the decisions they are forced to make within the context of disability. In this qualitative study we explored strategies adopted, factors influencing, and the impact of some of these strategies to manage the burden of medication cost among persons with disabilities. We interviewed 12 adults with spinal cord injuries living in Canada, using a general inductive approach to analyze the data. We found that before cutting back on medications due to costs, participants generally tried and sought help from the government, employers, and/or their prescribers to improve their drug coverage. The key factors that participants considered while making decisions on the strategies included the cost and perceived importance of medications, their financial status, other competing needs, and their relationship with the prescribers. While some of their efforts were successful, many participants were still not able to obtain their medications as prescribed. In those cases, patients resorted to rationing strategies such as cutting back on medications, other essential needs, or selling assets. These strategies had serious implications on their health, healthcare utilization, and quality of life.

## 1. Introduction

Despite the abundant literature on the treatment burden of chronic illnesses, there is limited research to understand the processes that underpin medication cost burden in general and especially among those with disabilities. The financial cost of living with a disability is substantially higher than that of living without a disability [1]. Our previous research has shown that the economic burden of medications faced by people with disabilities is higher than that experienced by their non-disabled counterparts. For people with disabilities, barriers to income and employment, higher susceptibility to health complications, complex medical regimens, additional health care costs, and the complexity of drug and social assistance programs perpetuate the risk of forgoing medications [2].

Prescribed medications for people with disabilities are often assumed to be covered under the provincial drug plans but many are not. This leads to a situation where people stop taking their medications (generally referred to cost-related non-adherence, CRNA) or adopt other rationing behaviors due to cost [3]. However, we do not know what strategies are used to cope with the cost burden of medication, how people are making medication rationing decisions, and the impact of rationing strategies on individuals, their families, and the healthcare system.

The literature that does exist has been mostly quantitative, generally examining prevalence or risk factors associated with CRNA. It includes older adults or individuals with chronic illnesses but is not specific to the experiences of people with disabilities [4]. Similarly, most commonly used theories that exist to define and understand medication non-adherence include the World Health Organization’s framework of general medication adherence (2003), Andersen and Newman’s healthcare utilization model (2005), and a model developed by Piette and associates (2006) [3,5,6]. However, all of these frameworks represent the factors that affect or increase someone’s risk of facing non-adherence to medications. None of these theories extend and depict the processes an individual with a disability might undergo or engage in after receiving a costly prescription.

To address this paucity of research, our research study explored the experiences of persons with disabilities managing their prescription drug costs. The specific objectives of this study were to find:

1. The strategies that participants adopted to manage their medication cost burden.
2. The factors that influenced an individual’s decisions to adopt those strategies.
3. The impact of rationing strategies on individuals.

## 2. Methods

***Design.*** It was a qualitative study wherein we adopted a general inductive approach to explore and describe the experiences of the participants with medication cost pressures. The inductive approach was adopted given the paucity of research to date on this topic [7]. The ethical clearance for the study was given by the Health Sciences Research Ethics Board (HSREB#912502) of Queen’s University.

***Context.*** The rising cost of prescription drugs has been leading to the rising incidence of CRNA in Canada [8]. The prevalence of CRNA is two to five times higher in Canada in comparison to other countries with universal healthcare [9]. Although Canada has a universal public health insurance program, it excludes universal coverage for prescription drugs [9]. People are either covered by the private plans, mostly provided by their employers; or provincial drug benefit plans that cover older adults (>65 years), people on social assistance, or people with catastrophic health needs [10]. As a majority of the people with disabilities remain unemployed or underemployed, they either have to forgo their medications because of a lack of private insurance or have to rely on provincial drug benefits to cover the costs of their medications. Even when medications are covered by the public drug benefit programs, the extent of coverage varies extensively and individuals have to share the costs of medications in the form of premiums, copayments, and/or deductibles. Calculated according to the individual’s income, these deductibles may range from 3% to 13% of annual household income that comes around $2000 to $9000 for a middle-income family and must be paid every year [11,12]. People who often work for small employers or are self-employed remain ineligible for the means-tested/income-based public drug programs [13,14]. 

The growing evidence on the cost-related barriers faced by many Canadians, especially those with low-income, poor health status, and disability, has raised many equity concerns, challenging the core principles of the Canada Health Act (i.e., universality and comprehensiveness). According to the 2017 Canadian Survey on Disability, 13% of all people with disabilities (*n* = 836,690) had unmet needs for prescription medications due to cost [15]. The prevalence of CRNA within people with disabilities is higher than that is found in general population in Canada (10%), suggesting that people with disabilities are more likely to face cost-related barriers to fulfill their medications.

***Sample.*** The sample for the study consisted of people with traumatic or non-traumatic spinal cord injuries (SCI) living in Canada. SCI affects around 85,000 Canadians living in the community [16]. The estimated lifetime economic burden associated with a traumatic SCI in Canada ranges from $1.47 million for a person with incomplete paraplegia to $3.03 million for one with complete tetraplegia [17]. People with SCI are high users of medications, taking between 5–14 different medications concurrently, often with complex regimens [18]. Therefore, individuals with SCI represented an ideal population of high users of medications for this study due to the chronic nature of their condition, the presence of multiple consequences of injury and the level of disability associated with an SCI [19,20].

***Sampling and recruitment.*** The sample for this study was selected from a subset of participants who reported facing cost-related barriers or were self-identified non-adherent participants in our first study [21]. In this first study, the overall sample was recruited with the help of community partners working with people with SCI including SCI Canada, Rick Hansen Institute, Canadian Spinal Research Organization, and other community-based organizations. An email invite was sent to their clients or the study information was posted on their e-newsletters or websites. To invite participants for this study, an email explaining the study details was sent to those participants who reported CRNA (*n* = 59) in the first study, of which 12 gave consent to participate for qualitative interviews. Written informed consent was obtained before interviews. Participants were offered $20 for their time and participation.

***Data collection.*** A semi-structured interview guide (Table A1) was developed based on the objectives of the study and the key findings obtained from the quantitative phase of the study [21]. The interview guide was pilot tested with two participants and revised in light of their responses. The interviews were conducted either in-person or by telephone, depending on the availability and choice of the participants, over a period of six months starting from August 2018 to January 2019. Interviews were conducted by the first author, who is a trained occupational therapist with the experience of qualitative research with individuals with SCIs. The interviews lasted between 40–60 min. The interviews were recorded using two audio-recorders and transcribed verbatim. To maintain anonymity and confidentiality, all personal identifiers were removed before data analysis.

***Data analysis.*** The coding process in inductive thematic analysis started with the preparation of raw data files after data cleaning; close reading of the text to understand the content; the identification and development of general themes and categories; the re-reading to refine the categories and reduce overlap or redundancy among the categories; and creating a framework incorporating the most important categories [22]. The first two authors (SG, MAM) coded the four transcripts independently to ensure inter-coder consistency and peer examination of the codes developed by the first author. The coding scheme was confirmed and corrected by the senior authors for any imprecise code definitions or overlapping of meaning in the coding scheme. Eighty percent of the total codes were identified within the first eight interviews while no new subcategories emerged after 10 interviews were completed. Two more interviews were conducted to confirm thematic saturation in data. These additional interviews verified that saturation is based on the widest possible range of data on the emerged subcategories. This process increased the comprehensibility of analysis and provided a sound interpretation of the data. The NVivo software was used to manage the data [23].

***Research rigor.*** Research rigor was ensured through an audit trail, member checking, and peer debriefing [24]. For an audit trail, a logbook was maintained that contained the notes on the data collection process, the analysis process, and the final interpretations. To ensure the credibility of the findings, the transcribed interviews and result summary were made available to all the participants in order for the participants to assess and correct the accuracy of their accounts. Out of 12, 10 participants agreed with the result summary and the rest did not respond to our request. The research team met at regular intervals to provide critical inputs on the research methods and researcher’s interpretation of meanings and analysis. The peer-review process involved deliberations and debriefing of the emerging codes, categories, and their relationship with the data. We used the consolidated criteria for reporting qualitative research (COREQ) checklist to report the methods and findings of the study [25].

## 3. Results

**Figure 1:**
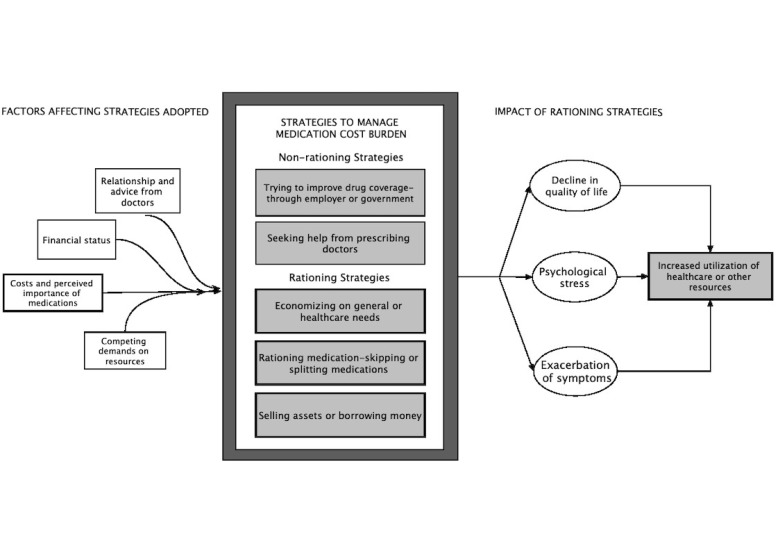
A conceptual model of patient approaches to manage medication cost burden.

The details of our sample are given in Table 1. Table A2 presents the coding scheme and definitions we adopted for the respective subthemes under each category. Figure 1 depicts the interplay of these subthemes which will be discussed further.

Overall, we found five key strategies adopted by our participants to manage the burden of medication cost. Out of these five, the first two were non-rationing strategies while the rest three were rationing strategies. Before cutting back on prescriptions due to cost, participants adopted non-rationing strategies. They proactively tried and sought help from the government, employers, or their prescriber to improve their coverage or opt for more affordable or less expensive drug substitutes. While some of their efforts to manage costly prescriptions were successful, many participants were still not able to take their medications as prescribed. In those cases, patients resorted to rationing strategies such as cutting back on medications, other essential needs or selling assets. The key factors that participants considered while making decisions on these strategies included cost and perceived importance of drugs, their financial status, the basket of financial resources they had to meet competing needs, and consultation with the prescribers. The rationing strategies caused financial stress and had serious implications on their health, healthcare utilization, and quality of life.

### 3.1. Strategies to Manage Medication Cost Burden

Five main strategies that were identified, in order of frequency, included:

#### 3.1.1. Trying to Access Public or Employer-Based Drug Benefits

A common initial strategy that participants described was obtaining drug benefits through public drug insurance or employer-based insurance. Some participants who were on disability support tried to improve their coverage through more generous public drug benefit programs, such as exceptional drug coverage which allowed access to drugs that are not usually listed or covered through the provincial drug formulary. This is illustrated by a participant who shared:

[Drug name 1] was not covered by the provincial Pharmacare, even though [drug name 2] was. I applied it under exceptional drug coverage, and they approved it on a yearly basis like I have to apply for it every year.

*[P/6]*

Participants described staying in current jobs to maintain employer-based drug insurance. Others who were self-employed at the time of the interview considered different employment to secure this type of drug coverage. A participant who had been self-employed in the past shared:

I am considering going back to work. Part-time, but if needed, full-time too. Right now, I am concerned about money to survive until retirement.

*[P/5]*

A few participants with a progressively worsening medical condition shared that they tried to maintain their jobs in order to remain eligible for employer-based drug benefits despite their worsening health situation. As a participant shared:

I had shift work, and it was very hard on me. In the last years, I tried to make it steady, but my body couldn’t take it. A lot of my disabled friends told me that I was practically killing myself to get those benefits.

*[P/9]*

Participants who tried to access more generous public drug benefit programs also shared many challenges they faced while doing so. Examples of the challenges include: the complex paperwork, eligibility requirements, the heavy deductibles, and the delay or wait times to get accepted for the drug program. These challenges are highlighted in the quote below, as the participant describes trying to access a public drug benefit program, known as trillium drug coverage:

Trillium, they have a huge [deductible] … you have to use up the deductible first, in order for it to be covered. My pills don’t work out to more than the deductibles, so it doesn’t apply to me. I tried to get on ODSP [Ontario Disability Support Program]. I have been turned down three times. We are (in) No Man’s Land.

*[P/3]*

Participants without jobs or other regular incomes had to wait for the approval from the Trillium program. The wait time spanned from a few days to sometimes several weeks. This resulted in some participants managing without medications for that wait time.

*It is frustrating as I have to submit [paperwork] first, to my insurance company, to my former employer, and then wait for them to get back to me, and then send all of this to Trillium. It’s such a delay. It is very frustrating because you may never know when you will get the money.*

*[P/7]*

It was evident from the participants’ accounts that while some of their efforts were successful, many participants were not able to improve their coverage.

#### 3.1.2. Seeking Help from Prescribing Doctors

The next common strategy that participants adopted to manage the burden of medication cost was to seek support from their doctors—either asking them to prescribe less expensive substitutes and alternatives or to help them enroll in a drug benefit program. Many times, doctors were able to help their patients access drug benefits or to prescribe less expensive and equally effective substitutes.

My doctors, all … my psychiatrist, my back specialist, and my family doctor they know I don’t have any benefits, so they always prescribe the cheaper alternative or over the counter alternative…or whatever, just for that reason.

*[P/2]*

However, in some cases, this strategy was not successful, especially when generic or other effective substitutes were not available on the public drug formulary or not covered by the public drug benefit program. A few participants shared:

My doctor was concerned about me. She would give me the samples for some medication because I had to cut back and she didn’t want to see me pay out of pocket. But with [drug name], it was too costly, and she couldn’t help me there. She tried to find cheaper substitutes or the generic versions, but they were of the same price range.

*[P/4]*

A participant suffering from a neurological condition shared how her doctor was cognizant of her financial situation as well as the time lag to get approval for the public drug benefits. As a result of this awareness, he applied for the benefits on her behalf well before her medical appointment.

My neurologist has to do that [application for drug benefit], and he is aware of the time-lapse. It has to be done every year. One year, he missed sending in the Section 8 form, and I was without drugs for a whole year. That would have been $4000–$5000 a month if I didn’t have coverage.

*[P/5]*

Although a participant’s relationship with their doctors played a major role in managing the burden of their drug cost, the participant had to initiate the conversation about the barriers they faced to purchase their medications. This is illustrated by one participant here:

My family doctor does not [ask me about medication costs]. Most of them do not ask that question. I have to bring it up. The urinary urgency one … it was about $300. I couldn’t get it for several months. I went to my urologist and told him that I couldn’t get it because I couldn’t afford it. At that point, he prescribed one that was less expensive.

*[P/5]*

#### 3.1.3. Economizing on General or Other Healthcare Needs

Participants who considered their medications essential shared that they either cut back on general needs such as food, clothing, car, leisure-related activities, or other healthcare supplies and needs—such as assistive devices. For example, a participant said:

I have stopped buying new clothes, and I shop at the thrift store. I have changed my spending habits, to make sure that I keep my health. Things like a gym membership, have also gone …

*[P/5]*

Participants’ accounts showed that when they had to choose among the general necessities such as food, clothes, heat, or car; clothing and leisure-related activities were the first to be eliminated or reduced. Basic or more important needs such as food and heat were last to go.

I could not have cut back on food, as you can’t survive without eating. But I didn’t buy clothes for years. We didn’t go on any trips forever! I don’t remember the last time we went on a trip. We just cruised along from paycheck to paycheck.

*[P/4]*

Most of these decisions were based on their health status, self-assessed health priority at the time, and their socio-economic status, as illustrated by this quote:

I have been needing new tires for my chair for two months (laughs). You have to make choices. Right now, my pressure sores are bad, and so that is my priority. I am not really going out much, stuck in bed, so the chair becomes my second priority.

*[P/9]*

#### 3.1.4. Rationing Medications

Participants also described taking smaller or less frequent doses; either postponing a refill of medication or stopping their medications for a period of time. For example, a participant shared “[I] had to postpone an antibiotic by a week or till I got paid” [P/2]. Also, having access to benefits, or other financial resources had a significant effect on the frequency or timing of rationing medications. A participant shared:

When I know I am down to the last few, if I have ten pills left today and I know I am not getting paid for three weeks, I’ll take one every other day. I’ll stretch it out till I get money.

*[P/7]*

A few participants also shared incidents when they were completely going without medications for a long period of time. To manage such situations, they chose between the two medications and sacrificed one to manage the other that was more important and costly to buy.

… Last month I could not afford [drug name] at the end of the month. So, I took my other pills, some of my three months pills I took a couple of days, so I could afford the [drug name]. Now, right at this moment, I am short again until my husband has the money coming in. Right now, I am taking one of one pill today, and one of the other tomorrow to get me through till the weekend.

*[P/3]*

Participants tried stretching-out medications slowly and gradually, depending on their perception regarding the effect of cutting back on medications and their financial situation.

… The recession hit, I was the last one hired, and first to go … For the next five years, I was doing temporary jobs. There were no benefits and I was paying out of pocket. That is when I started cutting back on the medication because I couldn’t afford to take it a hundred percent. It didn’t matter if I missed a needle. I thought I was going to die if I missed a needle. Turns out I was not, and I didn’t feel any difference. I thought if I missed two needles, then I would be affected. And like that, I missed three and four … I was taking medications for only 50 to 30 percent of the time. I was taking one needle a week, instead of every day.

*[P/4]*

#### 3.1.5. Selling Assets or Borrowing Money

This was the last alternative that participants sought to manage the burden of their mediation cost, especially when they had high additional disability-related expenses. They often tried to sell their house or car; use their available line of credit, or borrow money from friends. Participants in our study who were living alone or did not have a significant other along with more complex healthcare needs had a greater tendency to consider selling their assets. When asked, “how do you manage this burden”, a participant living alone after a recent divorce from her husband shared:

I have considered selling my house and going to an apartment. I have considered selling my car and getting something cheaper.

*[P/5]*

Participants who were living with their partners were generally more inclined to borrow money or use their line of credit, until the partner managed to find a job or other sources of financial support.

We got a huge line of credit on the house. We went into debt. We re-mortgaged the house and seriously considered selling the house. We just kept forking out this money and kept hoping we would win the lottery or something.

*[P/4]*

A few participants who neither had any assets to sell nor had a line of credit were more reliant on family members for financial support. Relying on the family for personal needs, especially for adults aged 30 to 60 in our study, resulted in a feeling of guilt or emotional stress, as illustrated by a participant here:

I am the lucky one. I get support on top of ODSP because my family can give me some money. It is an unpeaceful relationship. I don’t want to keep that relationship, but I have to because it is the only way I can survive on income that I get from ODSP.

*[P/1]*

### 3.2. Factors Affecting Decisions

This category involves factors that influenced participants’ decisions on the strategies adopted to manage the burden of their medication cost. Subthemes included:

#### 3.2.1. Cost and Perceived Importance of Medications

The first factor that influenced participants’ decisions regarding economizing on medications was the out-of-pocket cost of the medication. When participants had to choose between two medications for the same health condition, they often chose the ones that were more affordable to them. Sometimes, participants kept both medications, but they rationed on the one that was costlier while taking the other one as prescribed.

I usually continue the three months one, because it’s cheap. The other one I cut off, because its, you know, sixty-seven dollars a month. I’ll stretch out the other one.

*[P/3]*

In a few instances, participants chose between two medications that offer similar benefits, but have contrasting health risks and purchase price levels. For example, a participant who needed medication for sleep and pain shared that she chose the one that was less costly, though potentially harmful:

Given the choice of taking [drug name] and go to sleep, which is like a dollar, versus this other stuff which is $75 a bottle, I have to go with the highly dependent and habit-forming drug, unfortunately.

*[P/8]*

In situations when participants were taking multiple medications for different health conditions, or multiple medications for a single health condition without much cost-difference, they often chose a medication based on its perceived importance, or severity of a negative health consequence should the medicine be eliminated or reduced, in a calculation that considered the individual’s budget and broad range of medical issues.

It [drug name] certainly cannot be used interchangeably for the heart or blood thinning either. But I make sure I cover that. I have to cover. That means if I have to do without something else ... I have to do without something else.

*[P/1]*

In some incidents, decisions regarding choice of medications were often based on intuitive trial and error, especially when the cost of two medications did not differ much, or when participants were information-poor about adverse effects of choosing one medication over the other.

I am trying to decide what I can cut back. I am not really sure which one was good, because its trial-and-error. You try it, but you are not really sure if it is helping at all.

*[P/5]*

#### 3.2.2. Financial Status and Availability of Resources

The second important factor that drove participants’ decisions on the strategies to manage the medication cost burden was the availability of insurance or other financial resources at a particular point in time. For example, participants who did not have regular employment or income, such as individuals who were self-employed or students, had to cut back and make choices between medications and other needs until their financial situation or insurance status changed.

Then I was not a student, and so I had no coverage. I was self-employed. My insurance was $100 a month. As a self-employed person, I could not afford that. It really was a double-edged sword.

*[P/8]*

A participant who, at one point in her life, regularly took her medications as prescribed, had to eventually cut back because of her decreasing financial status.

There is a bunch of medicines I was taking over the years, which I could afford because the business was doing well. But because I have a fixed income and I am a single mom, I am going to have to cut back …

*[P/5]*

Another participant who was injured in an automobile accident and had insurance decided to pay for all of his medications out of pocket after insurance coverage was exhausted.

I was covered by the Manitoba Automobile Insurance till it ran out. It was a $200,000 fund. I have used it up over the thirty years. It ran out about three or four years ago. Now I pay for all of that [medications and other disability-related expenses] out of pocket.

*[P/6]*

#### 3.2.3. Competing Demands on Resources for Self and Others

A third factor that determined participants’ decisions regarding managing the burden of medication cost was the need for acquiring certain personal and inter-personal benefits, or the need to avoid or reduce certain personal and inter-personal threats. For example, some individuals, when their financial situation made them choose between medications and other basic needs such as housing, decided to manage without medications. For example, a participant shared:

I have to pay the bills first, otherwise, I don’t have a place to live! (laughs) For little things like medications, even though I need it, I workaround.

*[P/3]*

Some of the participants described their serious financial hardships due to medication cost burden when they had to choose between having food or looking after their children, over paying for and optimally taking their medications.

I have shared custody over our son … I eat a lot of peanut butter when he is not around. I put my own needs aside so that I can look after my son.

*[P/7]*

When they had more financial flexibility, they cut off on things that were relatively less important or desirable versus the ones that were essential, i.e., medications. For example, a participant who had a chronic neurological condition decided to cut off additional help for their household chores, as well as other complementary medications before cutting back on necessary prescribed medications.

I have a cleaner and somebody to mow my lawn … somebody to help with my groceries… those things have fallen off. All the complementary medicines have all been cut off.

*[P/5]*

In situations where participants had children or other dependents, they chose the needs of their children over the need for medications necessary to maintain their own health.

I would definitely choose his health over my health … I needed to cut back on some of my alternative medication so that I could buy him hockey equipment.

*[P/2]*

#### 3.2.4. Relationship and Advice from Prescribing Doctors

Participants’ decisions regarding rationing or balancing the cost burden of medications was also influenced by their relationship with their doctors. Some participants shared that if they had a good relationship with their doctors, they were generally able to discuss their financial situation and the limited ability to afford their medications. In the majority of cases, in doing so, they were able to find options to afford their medication and avoid non-adherence. A participant who was on disability support income and had her doctor’s support found alternative ways to fund her medications, or at least make rationing decisions in consultation with her doctor.

They [my doctors] try to adapt and try to get something else … they try to get special coverage ... I had doctors who would write several letters to make that special request … most times it is turned down by the government ... in the end, I will do without ... but doctors have been pretty good.

*[P/1]*

A few participants did not have a highly supportive relationship with their doctor and felt that they had no opportunity to discuss the medication cost burden with them. Sometimes they felt that doctors did not have enough time for candid discussions within a short consultation period. In such cases, participants made purchase decisions on their own, depending on their affordability, and/or the perceived importance of the medication. For example, a participant said:

I don’t get the opportunity to discuss anything with him [neurologist], because he just does his neurological examination and then I am dismissed. There is no opportunity for discussion.

*[P/11]*

Another participant shared that because she is served by a teaching hospital, this inhibits her ability to form a relationship with her doctor where she can discuss her finances and her inability to afford some of her medications.

I am at a teaching facility and every time I go, it is a different resident. I don’t find anybody who knows my file and I find educating them each time difficult. Sometimes that falls through the crack.

*[P/12]*

### 3.3. Impact

This category highlights the consequences of rationing strategies adopted by participants to manage medication cost burden. Subthemes included:

#### 3.3.1. Decline in Quality of Life

Not having enough money to pay for their basic medications and fulfill other basic needs had a negative impact on participants’ standard of living and overall quality of life. Financial hardships due to the burden of medication cost affected their security and freedom to live and enjoy life without worrying. They reported that they felt vulnerable, had a sense of scarcity, and were in constant stress managing the basic expenses within minimum financial resources. For example, a participant said:

I haven’t bought new clothes in a few years. In terms of grocery shopping … I get by with anything in the freezer or in the cupboard.

*[P/7]*

Sometimes their financial struggles were marginal while in other cases participants’ struggles were intense. Participants who were managing with a restricted income and who were navigating their chronic conditions alone frequently found themselves struggling with debt, having their credit seriously impaired, and using all of their savings:

I don’t do any recreational stuff. I don’t go out much either. I am still paying a mortgage, unfortunately, property taxes and all that great stuff. My whole cheque is eaten up… you could say I am living cheque to cheque.

*[P/9]*

#### 3.3.2. Exacerbation of Symptoms

Several participants quoted that discontinuing medications had a negative impact on their health condition and lead to the exacerbation of their symptoms. Some participants experienced the worsening of their symptoms much earlier and acutely while they were skipping doses. For example, a participant who was rationing between two medications for pain and depression shared:

When I skim the [drug name 1], I notice the depression increases, and if I skim the [drug name 2], then my pain symptoms increase. They are progressing, and if I don’t take [drug name 2], then the symptoms would get much worse, which would then stress me out even more.

*[P/7]*

Another participant who couldn’t afford her medications for a month experienced some of her symptoms worsening.

I haven’t had it for four weeks, because it wasn’t in my budget this month. I thought I would be okay, but I am not. I can see that I have white patches under my eyes, and I am bruising easily. Just four weeks off it, and I can see I am pale.

*[P/9]*

In other cases, participants noted the ill-effects of forgoing medications were evident much later when their health started deteriorating. For example, one of the participants who stopped one of her medications for a period of six months in the past was noticing a worsening of her symptoms and caught infections due to her compromised immunity:

A while ago, I wasn’t able to get my [drug name], as it cost $90 a package, which was way beyond my coverage … I did stop for a long time, as I could not afford it. It’s an obscene cost. Now, I am able to identify the symptoms and know what is going on … I have a lot of flare-ups, to the point that it covers a lot of my face. It’s very painful, and it brings on a Staph infection and that brings on a Strep infection, like flu. It can be very aggressive and painful.

*[P/8]*

#### 3.3.3. Psychological Stress

Many of our participants shared that along with their physical health, their mental health was affected by not having enough money to pay for their medications.

The psychological stress of it all is the biggest burden. It’s so not measurable, and it’s there every day. What are you going to buy? Where are you going to cut back? What do you need to buy for the future? Like, the wheelchair, I think what do I need to cut back to afford that? That is a lot of pressure.

*[P/12]*

The quote below is illustrative of a situation when participants were caught in a vicious cycle of stress which subsequently made their symptoms worse.

I didn’t have any [financial] support. I lost sleep, which causes immuno-deficiency, which causes stress, which causes cold sores, which causes loss of sleep. It was just a cycle.

*[P/10]*

In a few cases, participants’ accounts also showed that along with psychological stress, they faced emotional strain due to their restricted financial situation. For example, a participant who was on a disability support income had to ask her parents to buy some of her medications, which contributed to her feeling of humiliation and embarrassment.

I had to ask my parents for my meds, and it’s very, very humiliating and embarrassing. They are 90 and 91. Fortunately, they have been able to help me out but it causes a strain on them. I don’t like to do it, unless I absolutely have to.

*[P/7]*

#### 3.3.4. Increased Healthcare Utilization

The impact of the burden of the cost of medications was not just limited to patients’ physical and mental health but affected the public healthcare system too. Participants shared incidents when they had to visit the hospital more often or stay at the hospital for long periods in order to access drugs needed to manage their health condition. One of our participants was kept in the hospital for four extra days because the medication he needed was not covered by any drug insurance plan and was extremely costly to purchase.

When I was in the hospital, I was on an antibiotic for a bone infection … I was allergic to the IV, so I had to take a pill … that pill was 1000 dollars a day and only covered if you are in the hospital ... They wanted to release me from the hospital, but I had another four days to take the pill ... so I had to stay at the hospital for an extra four days … to get the 1000 dollar pill …

*[P/2]*

Another participant who lived in a smaller city and had no access to an interventional treatment involving a costly drug that she needed, was sent to the other hospital in a city nearby every week for her treatment. She was being reimbursed for all the costs involved.

I met a doctor in [city] who decides to send me to [name] hospital in [city]. This hospital … what it does is it used a freezing element to freeze my nerves at the back and my neck to relieve some of my pain. It did help to some degree. But that meant every single week I am on the bus staying at a hotel for one night. Incredibly costly to the healthcare system

*[P/1]*

Overall, participants felt that cost savings to the healthcare system made through restricting drug coverage would be negated. People who stop taking medications or cutting back on food will ultimately end up using the healthcare system more frequently.

If a person is not able to access nutrition, they will use the healthcare system more, and that is costly for the government ... same for the provincial government, if the ODSP is cut, then people will need ADP [assistive devices program] more. If it doesn’t come out from one pocket, it will come out of another.

*[P/10]*

## 4. Discussion

To our knowledge, this is the first qualitative study in Canada that has explored patients’ experiences with managing the burden of the cost of their medications within the context of special vulnerable populations. We found that before deciding to stop taking medications, participants tried a few non-rationing strategies to access their essential medications (Figure 1). Participants proactively tried and sought help from different potential sources including government, employers, or their doctors. They either tried to improve their coverage through public drug benefits or opted for less expensive drug substitutes. However, while some of these efforts were successful, many participants were still not able to fulfill their medication needs without facing financial hardships. In those cases, patients resorted to other strategies that had serious implications for them. For example, they economized on other health-care or basic needs such as food, clothing, or leisure-related activities. Some considered selling their assets or borrowing money to manage their medications. In some instances, participants used those strategies in combination while in some only one of these strategies were used.

Even when individuals were covered through provincial drug benefits, they faced many systemic barriers including complex paper-work to meet eligibility requirements, heavy deductibles, restrictions on public drug formularies, and long wait times for getting approvals, all of which resulted in forgoing medications due to cost. These results support the findings from other studies that suggest that just having insurance does not safeguard individuals from facing financial hardships completely [26,27]. This is specifically true for the people with disabilities who, despite receiving subsidized care, face deprivation due to extra costs of living with a disability and may find even small co-payments difficult to manage. Even among people with disabilities, we found that our participants were on a continuum of facing financial stress due to medications. Participants who were at the extreme end of financial stress often were living alone, had higher overall healthcare costs, poor financial resources, and social support.

The factors that participants considered while making rationing decisions included drug costs, their perceptions around the negative consequences of skipping medications, the basket of financial resources they had to fulfill their needs and that of their family, and the relationship with their doctors. Evidently, while most of these decisions were solely made by patients themselves, some decisions were guided or sanctioned by their doctors. Awareness by the medical professionals of the adherence barriers in patients has long been identified and proven as one of the effective strategies to protect against the non-adherence of medications [28]. To do this effectively, doctors need to be cognizant of the potential burdens for all patients and help those in need to navigate these barriers through care coordination. For example, there have been guidelines to help prescribers consider the generic and branded names of drugs, their market cost, and availability of insurance coverage when treating their patients with chronic medical conditions [29].

The other main finding of the study was that financial barriers to medications not only affected patients’ physical health, but also impacted their overall quality of life, and highlights the costs and inefficiency impact to the healthcare system. Patients were trapped in a vicious cycle of stress and financial scarcity which led them to cut back on basic needs or ask for money from friends or family. These stressors caused exacerbation in their symptoms which ultimately caused them to use more healthcare services, both related and unrelated to their medications. Studies involving other populations have also found that treatment burden operates in a cyclical manner and often has a catastrophic impact on patients, and their households which further trickles down to healthcare system [30,31,32]. Studies from Canada, in particular, demonstrate that sometimes doctors have to keep their patients in the hospitals to allow access to drugs that are costly and not covered outside the hospitals [33,34].

### 4.1. Policy Implications 

This study has identified a complex array of choices made by the patients to access prescription medications. Yet they were not fully successful in improving the access. This lack of access adversely impacted their health and healthcare utilization. Thus, the issue needs to be addressed at the level of the healthcare provider, the healthcare system, community, and social services, employment, and insurance policies.

Recently, in response to the report from the Standing Committee on Health, the Canadian government announced the formation of a national advisory council on the implementation of an affordable Pharmacare in the 2018 budget [35]. The report of the advisory council that was recently released, recommended a universal single-payer public prescription drug system for Canada. This recommendation has given high hopes to Canadians, though how the implementation of these recommendations would benefit people with disabilities, in particular, remains elusive.

We propose that as Canada contemplates national Pharmacare, there are some short-term and long-term solutions that might be considered. In the short-term, prescribing healthcare professionals can play a key role by being cognizant of their patients who are at risk of out-of-reach medication costs i.e., individuals without regular income or employment, with poor health status and disabilities, and those having high healthcare and prescription medication needs. Healthcare professionals can minimize this risk by choosing medications that have better coverage or lower costs for their patients. Healthcare professionals also need to let the patients define what out-of-reach financial costs are, as for some patients, even $50 per month can be out-of-reach. In the longer run, we propose that the government look into eliminating the systemic barriers of drug insurance arrangements (i.e., access and cost). The processes to get coverage through the provincial drug benefit programs should be simplified (e.g., yearly forms to be filled out by physicians is inefficient and burdensome to patients and physicians, especially for disabilities that are life-long). Public drug formularies should provide generous coverage to the drugs that are required by people with diverse disabilities. The heavy deductibles to meet eligibility requirements for exceptional drug coverage should be reconsidered and wait times to get approvals should be minimized.

Our results also showed that the minimum income afforded to individuals on disability requires that they ration their funds to access other over the counter or complementary medications. Revisiting a minimum basic income for individuals with a disability would be important. Employers may also ensure that disabled employees are appropriately accommodated, for example, with part-time work without restricting their access to group health benefits. These solutions may not only help Canadians with disabilities but also any Canadian who faces a financial barrier to access their necessary prescriptions.

### 4.2. Study Limitations 

This study has several limitations. Since our sample was small and comprised of people living with SCIs in Canada, our results may have limited generalizability to other populations or context. It would be interesting to compare our results with studies that explore the burden of medication cost for people with other types of disabilities. Because we used a qualitative approach at one point in time, we could not explore how these experiences changed over a period of time or with advancing age or disability. Although Canada is a multi-lingual country, the interviews were only conducted in English due to the feasibility with the research team. Conducting interviews in other languages might have allowed us to capture other diverse views. Some of the interviews were conducted telephonically which might have led to the absence of visual cues from participants accounts. Though, telephone interviews are judged to be equally rich, detailed, and of high quality as the face-to-face interviews [36]. Also, as the interviews were primarily conducted by the first author, the results might have been influenced by her own bias and experiences. However, we employed a number of methods to maximize rigor, including member checking, peer review, rich description, and maintaining field notes.

## 5. Conclusions

The burden of medication costs among people with disabilities is overwhelming. Results of our study provide an in-depth evaluation of the processes underpinning as well as the after-effects of rationing or forgoing medications due to costs, for people living with complex disabilities. Despite the efforts made by the participants to improve coverage and fulfill their medication needs, many had to face serious hardships to manage their medication cost. These stressors exacerbated their symptoms which ultimately caused participants to use a greater range of healthcare services more frequently, both related and unrelated to their medications. These findings have important implications for healthcare policymakers to minimize systemic barriers within drug insurance programs, and prescribing healthcare professionals to be vigilant for patients who may encounter financial barriers fulfilling medications as well as future researchers to identify medication cost burden among people with other types of disabilities.

## Figures and Tables

**Table 1:** Participant characteristics (*n* = 12).

Participant Characteristics	*n*/Median (q1, q3) *
Age (years)	57.5 (46, 58.25)
Females	8
Relationship status	
● Married or in a relationship	5
● Single, divorced or never married	7
Education	
● Up to high school	2
● College degree or certificate	7
● University degree and above	3
Work status	
● Employed	2
● Retired	1
● On disability income	8
● Unpaid disability and unemployed	1
SCI-related characteristics	
● Traumatic	6
● Paraplegia	9
● Incomplete	6
● Time since injury (years)	20 (10, 28.5)
Number of medications ^1^ (median)	9.5 (5, 13)
Monthly out of pocket cost of medications ^1^ (CAD)	316.5 (181.25, 398.75)
Type of insurance ^2^	
● Public drug benefit program	8
● Employer based insurance	4
● Family based insurance	1
● Other	1
● No insurance	2

^1^ Include both prescribed and over-the-counter medications; ^2^ The numbers do not add up to 12 as a few participants had more than one type of drug insurance coverage. * q1 and q3 refer to the first (25th percentile) and third (75th percentile) quartiles respectively.

## Appendix A

**Table A1:** Interview guide.

You indicated in your survey that you sometimes have trouble paying for your medications, and that you even sometimes don’t take your medications as prescribed because of the cost. In this interview, I’d like to ask you more about that.
1. How often does this happen? When is the last time it happened?
2. Does it happen more often with some medications than with others?
3. How do you decide what to take and what not to take, or how to adjust your dosage to save money?
4. Do you tell your doctor when you are doing this? Do you and he/she discuss the cost of the medications prescribed?
5. What do you do to manage or cope with medication cost burden?
6. What about other supplies and non-prescription medications—do you ever economize on those?
7. What is your priority for spending on things for your or your family’s health?
8. How do you think your health has been affected by not taking your pills as prescribed, or by other compromises you have had to make because of costs?

**Table A2:** Coding scheme.

Strategies	Factors Affecting Decisions	Impact of Rationing Strategies
Strategies that participants adopted to cope with medication cost burden	Factors that influence participants’ decisions to adopt rationing strategies	Consequences of forgoing medications due to cost or financial stress caused due to medications 7
1. Trying to access public or employer-based drug benefits	1. Cost and perceived importance of medications	1. Decline in quality of life
statements when participants tried to go back to work, access public drug benefits, or other social assistance to manage medication cost burden	statements where participants decided to choose to stop or continue taking a medication due to its cost and on their perceived importance or severity of their health condition or a health situation for which they were prescribed medication	statements when participants indicated that medication related financial burden reduced their general well-being or life satisfaction for them or their family members
2. Seeking help from prescribing doctors	2. Financial status and availability of resources	2. Exacerbation of symptoms
statements where participants shared that they requested their doctors or pharmacist for less expensive substitutes such as generics or over-the-counter medications	statements when participants stopped or rationed on medications depending on their financial status or availability of financial resources at that particular time	statements when participants indicated that rationing on medications due to cost has led to increase or worsening of their symptoms or experienced pain
3. Economizing on general or healthcare needs	3. Competing demands on resources for self and others	3. Psychological stress
statements where participants shared that they cut back on other healthcare supplies or needs such as assistive devices, or general needs such as food, clothing, car, or leisure-related activities to manage medication cost burden	statements that reflected that participants made decisions to stop or ration on medications based on their priorities for their children, other significant health needs or basic life needs	statements when participants indicated that medication related financial burden caused mental or psychological stress
4. Rationing medications	4. Relationship and advice from prescribing doctors	4. Increased healthcare utilization
statements where participants shared that they decided to stop a medication temporarily or for a long duration, or taking smaller or less frequent doses, or postponed refill of a medication due to high cost	statements when participants stopped or rationed on medications in consultation with their doctors or lack thereof	statements when participants indicated that rationing on medications due to cost has led to increase in use of other health services such as more doctor visits, or even hospitalization
5. Selling assets or borrowing money		
statements that reflected participants tried to sell their assets or borrowed money to manage financial burden due to medications and other healthcare costs		
